# Occurrence, treatment and pathogens involved in mastitis on a commercial German dairy farm: A retrospective study from 2012 to 2021

**DOI:** 10.5455/javar.2024.k837

**Published:** 2024-12-27

**Authors:** Tina Kabelitz, Olivier Basole Kashongwe, Marcus Doherr, Ulrich Nübel, Christian Ammon, Pablo Silva Boloña, Orla Keane, Thomas Amon, Barbara Amon

**Affiliations:** 1 Department of Engineering for livestock management, Leibniz Institute for Agricultural Engineering and Bioeconomy e.V. (ATB), Potsdam, Germany; 2 Department of Veterinary Medicine, Institute for Veterinary Epidemiology and Biostatistics, Freie Universität Berlin, Berlin, Germany; 3 Department of Microbial Genome Research, Leibniz Institute DSMZ-German Collection of Microorganisms and Cell Cultures, Braunschweig, Germany; 4 German Center for Infection Research (DZIF), Braunschweig, Germany; 5 Braunschweig Integrated Center of Systems Biology (BRICS), Technical University, Braunschweig, Germany; 6 Animal and Grassland Research and Innovation Centre, Teagasc, Fermoy, Ireland; 7 Animal and Bioscience Department, Teagasc, Dunsany, Ireland; 8 Department of Veterinary Medicine, Institute for Animal Hygiene and Environmental Health, Freie Universität Berlin, Berlin, Germany; 9 Faculty of Civil Engineering, University of Zielona Góra, Zielona Góra, Poland

**Keywords:** Bovine, udder inflammation, cow, milk, livestock, AMS

## Abstract

**Objective:**

Mastitis is the most common and costly dairy cow disease worldwide. We performed an intensive analysis of mastitis prevalence, pathogens, and treatments using retrospective data from a commercial dairy farm in Germany to estimate the severity of mastitis in the commercial production system and to give on-farm insights.

**Material and Methods:**

Milking system data and cow-individual data were collected over 9 years (2012-2021). A resilient amount of data from 1537 cows, >1,000 mastitis infections, 1901 pathogens, and 5729 treatments have been analyzed.

**Results:**

Mastitis occurrence was highest in summer (45.0%), in first lactation (51.1%), and in the late lactation stage (36.7%). The relative mastitis frequency increased sharply with a high lactation number (>7). The leading pathogens causing mastitis were coagulase-negative staphylococci (28.3%). Approximately 25% of mastitis cases were treated with non-antibiotic medicine and 75% with antibiotics. For the latter, cephalosporins and aminoglycosides were the most administered. The average mastitis treatment duration was 3.48 days. During the study time, the farm changed from a conventional milking system to an automatic milking system in 2015, which has not negatively affected the number of recorded mastitis infections.

**Conclusion:**

This case report gives detailed insights about mastitis incidences gained under practical conditions. Novel information about mastitis drug usage and duration is presented. Potential mastitis risk factors identified from the results of this study were the summer season, first or >7 lactation(s), and the late lactation stage.

## Introduction

Mastitis (inflammation of the mammary gland) continues to be the most common and costly disease in dairy milk production and is of worldwide relevance [1–3]. It negatively affects the farmer’s economic profit and is an important animal health and welfare issue. Mastitis is associated with negative consequences for animals, farmers, and the environment, such as reduced milk quality, increased labor, and veterinary costs; increased use of antibiotics, and thereby, selective pressure in favor of antibiotic-resistant microorganisms; as well as suffering and culling of cows [4]. Additionally, milk yield is reduced during mastitis [5–7]. The milk losses range from 0.07 kg per quarter of milking to 1.4 kg [5]. Together, this results in economic costs and losses of approx. 125€ per cow per year and accounts for roughly 40% of the total direct costs of common dairy production diseases [4,8]. Mastitis incidences are typically between 30% and 50% per cow per year [1,9]. Mastitis is classified into subclinical cases (no observable abnormalities of the cow and the milk, but a lower milk yield and a higher milk somatic cell count) and clinical cases (clear abnormalities observable of cow and/or milk) [4,10]. Subclinical mastitis is the most common type and more difficult to detect than clinical mastitis. Therefore, it is of higher economic relevance [11].

The predominant bacterial species and groups, causing mastitis in >80% of all cases, are* Escherichia coli, Streptococcus uberis, Staphylococcus aureus, *coagulase-negative staphylococci (CNS), *Streptococcus dysgalactiae, *and* Streptococcus agalactiae* [8,12–14]. However, the dominating mastitis pathogens vary depending on production systems, climate conditions, and hygiene standards. Therefore, mastitis pathogen prevalence can vary even on the local scale. Mastitis pathogens can be grouped into environmental and contagious [4]. Environmental pathogens are part of the normal microflora and are present in the cow environment (skin, bedding material, dirt, and so on) [15]. The risk of causing an infection is dependent on their concentration in the environment and the susceptibility of the cow. Environmental pathogens invade the mammary gland when the teat canal is open after milking or after damage. In contrast, contagious pathogens are adapted to survive within the mammary gland, and they spread from cow to cow primarily during the milking process [16]. Some mastitis pathogens, e.g.,* Strep. uberis* and *Strep. dysgalactiae,* act as not completely environmental or contagious, and intermediate types are described [17,18].

Currently, due to high hygienic conditions in milk production facilities, the majority of mastitis cases are caused by environmental pathogens [19,20]. Mastitis control and treatment are the two major reasons for antibiotic usage in dairy cows and enhance the risk for the emergence of antibiotic-resistant microorganisms and their spread in the environment [21–23]. Therefore, it is a main issue in dairy farming to prevent mastitis infections and to detect them early to reduce antibiotic usage. Mastitis is a complex and multi-factorial disease that can cause diverse disease symptoms (from none to life-threatening) depending on cow-individual factors and the pathogen type. It is known that several host and environmental factors influence the susceptibility of cows to mastitis. Host factors include, e.g., cow age, parity, lactation stage, genetics, mastitis history, mean somatic cell count (SCC), mean milk yield, and teat anatomy [16,24]. Environmental factors influencing the mastitis probability are weather conditions (season), management practices (floor type, use and type of bedding material, frequency of bedding exchange, milking method, and so on), and general hygienic conditions, especially around the milking process. Previous studies showed that udder health was negatively affected by a change from conventional milking to an automatic milking system (AMS) and generally remains so in AMS systems [25].

To the best of our knowledge, available data about drug usage (type, duration, and variability over the years) for mastitis treatments are limited. In this study, large-scale and detailed retrospective farm-derived mastitis data gained under practical conditions, which are rarely published so far, were analyzed. Data about mastitis recurrence rates, drug usage, and treatment duration are especially important. The outcomes of this case report will help to estimate the severity and consequences of mastitis in the commercial production system and give novel insights into the on-farm mastitis situation.

## Materials and Methods

### Description of farm and herd

Data used in the study originated from a commercial dairy farm with a herd size of approx—230 cows in Northern Germany. Cows were kept in a naturally ventilated barn with a free cow traffic routine and lying cubicles. Cubicles were bedded with a mixture of straw and lime. Lying areas were cleaned twice a day, and the whole bedding was exchanged once a week. On hot days, the cows received artificial cooling by ventilators and air pipes. Cow-walking areas were built of concrete floors that were cleaned once per hour by an automatic manure scraper. At the end of the scraper, the ground was equipped with slatted floors. Cows were fed two times a day with a total mixed ration. The remaining feed was moved into position five times per day. Concentrate was provided during milking, and the amount was adapted to the cow's milk yield. In 2015, the farm changed from conventional milking to an AMS. Cows were milked approx. 3 times per day by a Lely Astronaut A4 AMS (Lely, Maassluis, Netherlands). The herd consisted of Holstein-Friesian dairy cows from lactation 1 to 9 with an average milk production of 29.8 l per cow per day. The maximum milk yield of cows varied between 50 and 60 L per cow per day. Every cow had a necklace for identification at the AMS, recording of rumination, and locomotion, and to predict the estrus phase. Heifers were inseminated at approx. 15 months and calved first with approx. 24 months. Heifers and dry cows were kept on pasture land. Calving events took place in a straw-littered barn evenly throughout the year.

### Mastitis prevention

Mastitis preventive measures included a farm with a high hygienic standard through extensive cleaning and disinfecting of the AMS at least three times per day, short automatic cleaning and disinfecting of the AMS after every cow, udder cleaning before milking with automatic brushes (cleaned and disinfected after every cow), and teat disinfection by spraying iodine solution after every milking. Udders and tails were regularly clipped. Routinely, the daily SCC for every cow is estimated by the AMS based on a modified California mastitis test. Monthly milk control and composition determination were performed for each cow by a certified milk laboratory. Before the dry period, quarter milk samples of each cow were collected and submitted for bacteriological analysis by a certified laboratory. According to bacteriological and SCC results, quarter-selective dry cow therapy was performed using teat sealant and/or antibiotics, if necessary.

### Mastitis identification

Mastitis in this study was identified by abnormalities in the milk confirmed by a visual inspection of the first milk strips (clinical mastitis), done by a herd’s person, or by an elevated milk SCC (subclinical mastitis) identified with a manual milk viscosity test (California mastitis test). During milking, the AMS recorded several quarter milk parameters, including milk color (bloody milk), conductivity, temperature, yield, SCC, fat content, protein content, milking flow, and so on. The AMS generated an automatic alarm to the herd manager when milk conductivity, milk temperature, color, and/or SCC were elevated or when the cow did not come for milking for >24 h. For cows for which an alarm was generated, the foremilk of each quarter was visually checked, and a California mastitis test (also called a “Schalmtest”) was performed by a herdsperson. When visual abnormalities in the milk or an increased SCC were observed, a sample for bacteriological analysis was sent to a mastitis laboratory, and in consultation with the veterinarian, a mastitis treatment was initiated immediately.

### Mastitis treatment

Depending on the mastitis type (clinical or subclinical) and the severeness of symptoms, antibiotics (cephalosporins, aminoglycosides, fluoroquinolones, or penicillin) or alternative medicine (anti-inflammatories, vitamins, minerals, or homeopathic) were applied. Until clinical cure, the affected cow was separated from the herd to prevent the spreading of the infection and was kept in a straw-bedded pen. During treatment, the cow was milked twice per day in a conventional milking parlor. Milk of antibiotic-treated cows was discarded. After the disappearance of sickness symptoms and the drug withdrawal period, the cow returned to the herd. Cows with chronic mastitis (>3 infections within 6 months) were culled.

### Data analysis

Data from 1,537 cows, 1051 mastitis infections, 1901 mastitis pathogens, and 5,729 mastitis treatments were collected and analyzed over nine years (2012–2021). The following parameters were used for data analyses: season of mastitis occurrence (spring, summer, autumn, winter), lactation number (0-9), lactation stage (before calving, early, mid, and late), number of mastitis cases per cow per year, causative mastitis pathogen (no growth, CNS, *E. coli, Staph. aureus*, yeasts, *Strep. uberis, Strep. dysgalactiae, Strep. agalactiae*, or others), drug type used for mastitis treatment (antibiotics and non-antibiotics), mastitis treatment duration (in days), and total number of mastitis cases. The following data processing techniques were used for data analyses: removal of missing values and duplicates, grouping of mastitis treatments per antibiotic class and non-antibiotic group, and the generation of the variable “season” according to calendar months. All processing was done in Python 3.8.8, using pandas and numpy libraries. Numerical descriptive analyses (frequencies and percentages) were generated using the crosstab package of pandas. Statistics such as frequencies and percentages were used to assess the seasonal distribution of mastitis occurrence, seasonal herd SCC, lactation number, lactation stage, pathogen frequencies, recurrent mastitis cases, mastitis treatment duration, and medication. Significant differences between means were determined by the Student's *t*-test (*p* < 0.05). The mean variability of data was calculated by standard errors of the mean.

## Results and Discussion

### Mastitis prevalence

The herd we studied showed an overall mean mastitis prevalence of 38% per cow per year (data obtained from monthly milk control reports, not shown), which is in agreement with typical estimates of mastitis infection rates of 30%–50% per cow and year [1,9].

### The influence of season and year

**Figure 1.**
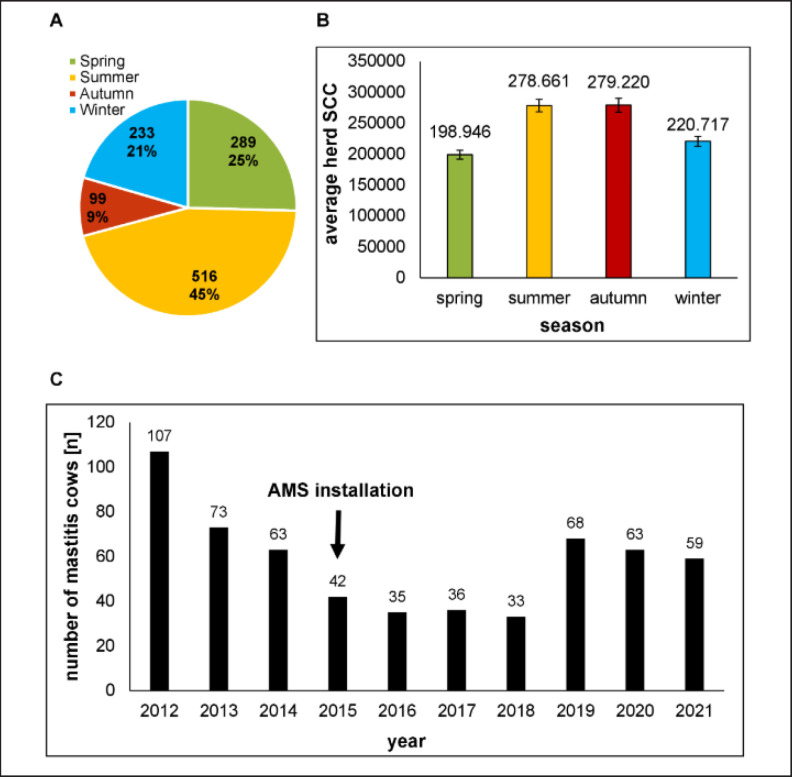
A) seasonal mastitis frequencies (absolute numbers and percentages), B) seasonal average herd SCC. Error bars show the standard error of the mean. C) number of mastitis cows over the monitoring period from 2012 to 2021. AMS: automatic milking system.

We observed that 45% of all recorded mastitis cases occurred during the summer (Fig. 1A). Mastitis pathogens detected with a peak in the summer were yeasts and *Staph. aureus*. Mastitis rates observed in spring and winter were 25% and 21%, respectively. Mastitis-causing pathogens with seasonal peaks were *E. coli* in spring and *Strep. dysgalactiae *as well as* Strep. agalactiae *in winter. The lowest mastitis frequency was detected in autumn (9%). Here, *Strep. uberis* mastitis was more abundant than in other seasons. CNS mastitis was equally distributed over the whole year, and CNS were the most frequently isolated bacteria. The highest herd SCC levels were observed in summer (278.661 cells per ml milk), in correlation with the highest seasonal mastitis frequency. However, SCC values were high also in autumn (279.220 cells per ml milk), where we detected the lowest prevalence of mastitis during the year (Fig. 1B). The lowest average herd SCC was observed in spring (198.946 cells per ml milk). Resulting in a yearly percentage variation of mean herd SCC of a maximum of 28.8%. These results agree with former studies, where the highest mastitis rates were observed in summer, compared to spring and winter [24]. The reasons might be the high temperatures in summer and thus the higher proliferation rate of environmental pathogens or their transmission with insects, mainly flies, which are most abundant during summer. Furthermore, cows are sensitive to heat stress with negative effects on their welfare, health, and performance [26,27]. Heat stress arises when the endogenous heat production of a cow is greater than its capacity to lose heat. It is not only dependent on the environmental temperature, but also on the humidity, solar radiation, wind speed, and cows' susceptibility [28]. Heat stress results in a higher body temperature, increased respiration and heart rate, less feed intake, less milk production, and reduced lying and rumination times [29–31]. Heat stress causes a reduction of the cow's general health and decreases the immune system function, which makes it more susceptible to pathogens and diseases during summer.

When analyzing the number of mastitis cows per year over the study period (Fig. 1C), we counted a maximum of 107 mastitis cows in 2012, the first year of analysis. From then on, the number was strongly decreasing for 4 years (2012-2016) and stabilized in 2016-2018 with 33-36 mastitis cows per year. In 2019-2021 the numbers of infected cows per year increased to 59-68. The AMS was installed in 2015, which did not cause any rise in recorded mastitis cows, although it is documented that often problems appear following the conversion from conventional milking to AMS [25]. Results from Hovinen and Pyörälä [25] showed that udder health is negatively affected by a change from conventional milking to AMS and generally remains so in AMS systems. This was not observed in our study.

### The influence of lactation number

**Figure 2.**
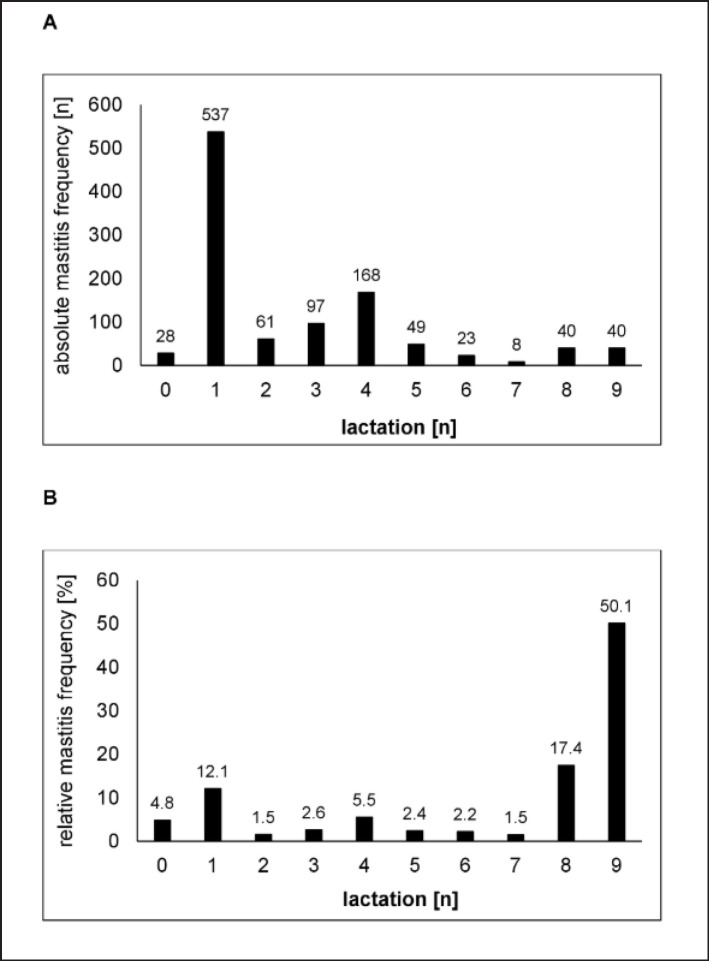
Mastitis frequencies according to lactation number. A) absolute frequencies (number of mastitis infections), B) relative frequencies (% distribution of mastitis cases in every lactation). Mastitis in lactation 0 refers to heifer mastitis within 14 days before first calving.

When analyzing the occurrence of mastitis in correlation with lactation number, it is necessary to distinguish between absolute and relative mastitis frequencies within a herd. Because age distribution within a typical dairy herd is unequal and consists of mainly young cows with low or median lactation numbers (0-5), compared to old cows with high lactation numbers (≥ 7). The average productive life of a Holstein-Friesian cow in Germany is 2-3 lactations (37 months), meaning that a dairy herd is composed mostly of young animals [32]. When analyzing absolute mastitis frequencies, we recognized that most mastitis cases (537 of 1051% = 51.1%) occurred during the first lactation (Fig. 2A) because the first lactating cows make up the largest proportion in the herd. A second peak for absolute mastitis infections (*n* = 168) was recorded in the fourth lactation. The lowest absolute mastitis frequencies were observed for cows in the seventh lactation, followed by the sixth lactation and heifers (0 lactations). Heifer mastitis means an infection in the 14 days before calving. Looking at relative mastitis frequencies, the distribution looks different (Fig. 2B). Relative to the number of cows within the same lactation, the highest number of cows that developed mastitis were in the ninth lactation. 50.1% of all relative mastitis cases were observed in the ninth lactation, followed by the eighth lactation (17.4%). Therefore, a sharp increase in mastitis risk after the seventh lactation can be concluded. The relative mastitis frequency in the first lactation was elevated as well (12.1%), but not as abundant as for the absolute numbers.

The lowest relative risk for developing mastitis was for cows in their second and seventh lactation (both 1.5%). It was previously reported that the mastitis frequency increases with the number of parities and that old cows with many lactations have a 24% higher probability of developing mastitis than young or medium-old cows [4,24,33]. Cows with a high calving number (>6) have been reported to develop mastitis 30% more often than cows with few calving (1–3). This is due to the trend that older cows with many parities show a wider or permanently open teat canal as a result of frequent milking. Due to the lower performance of this natural udder protection mechanism, it is easier for pathogens to invade the teats of old cows. This observation is confirmed by our study when focusing on relative mastitis frequencies. However, absolute mastitis frequencies are of higher practical relevance and were highest in the first lactation. Maybe reasoned by CNS being the farm-leading mastitis pathogens (Fig. 5). CNS are known to be the most prevalent cause of subclinical intramammary infections in heifers and primiparous cows [34].

### The influence of the lactation stage

**Figure 3.**
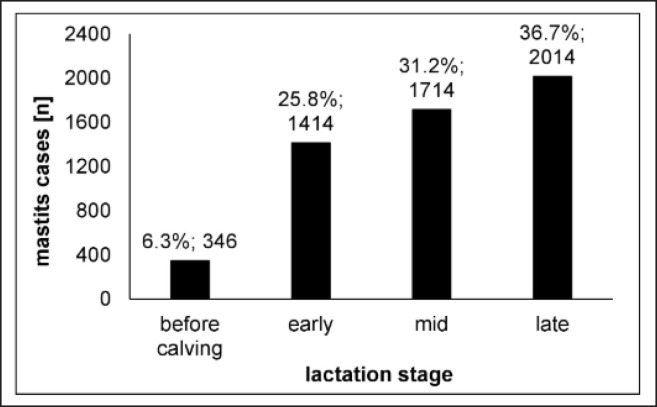
Mastitis frequencies according to lactation stage before calving, in early (lactation months 1–3), mid (months 4–6), and late lactation (months ≥7).

Analysis of mastitis infections during different phases in lactation revealed an increasing frequency over the lactation period (Fig. 3). Mastitis frequency was 25.8% in early lactation (lactation months 1–3), elevated in mid-lactation (months 4–6) (31.2%), and highest for late-lactating cows (months ≥7) (36.7%). The lowest mastitis occurrence was observed before calving (6.3%). The distribution observed for this farm is a bit uncommon because, in the literature, the higher mastitis risk is supposed for early lactating cows (months 1–3) [4,24,33]. The high incidence of mastitis during the early lactation phase, especially directly after calving, is supposed to be due to a negative energy balance, meaning that the cow needs more energy and nutrients than it can consume. A negative energy balance leads to immunosuppression, associated with increased oxidative stress and low antioxidant defense, causing a higher susceptibility to diseases [4]. This is particularly true for high-yielding dairy breeds (e.g., Holstein-Friesian) under intensive production conditions. In the current study, we could not confirm the observation of the highest mastitis frequency in the early lactation state.

### Recurrent mastitis

**Figure 4.**
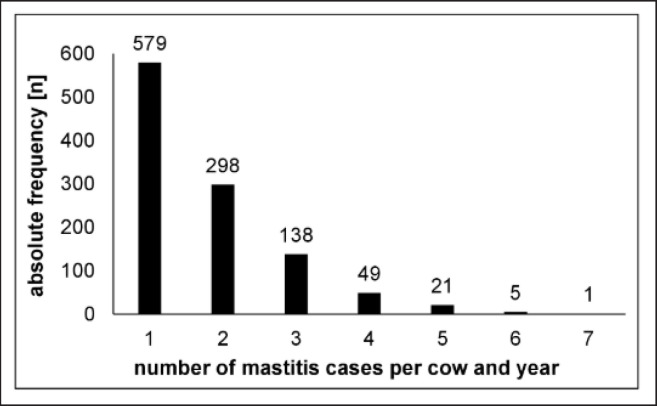
Frequency of mastitis recurrence.

We calculated the number of mastitis cases per cow per year to do some estimations of the recurrence of mastitis (Fig. 4). In 579 cases (53%), one mastitis per cow per year was observed. In 512 cases (47%), recurrent mastitis (>1 mastitis infection per cow per year) was determined. They showed that mastitis recurrence is a serious phenomenon in milk production. The observed frequency of mastitis recurrence is in total agreement with other studies, which observed a recurrence rate of approx. 50% for clinical mastitis [35,36]. Reasons for mastitis recurrence can be either persistent or novel infections. Persistent mastitis infections can be caused by unsuccessful therapy, e.g., due to too low antibiotic concentrations at the site of action, antimicrobial resistance of the pathogen, pathogen biofilm generation (e.g., by *Staph. aureus* and *Strep. agalactiae*)*,* or intracellular growth of the pathogen [18]. Wente et al. [35] investigated 445 recurrent mastitis cases and confirmed 145 (32.6%) to harbor the same pathogenic species and 49 (11.0%) caused by even the same species strain. Identifying the same species strain makes a persistent mastitis infection highly likely.

### Mastitis-causing pathogens

**Figure 5.**
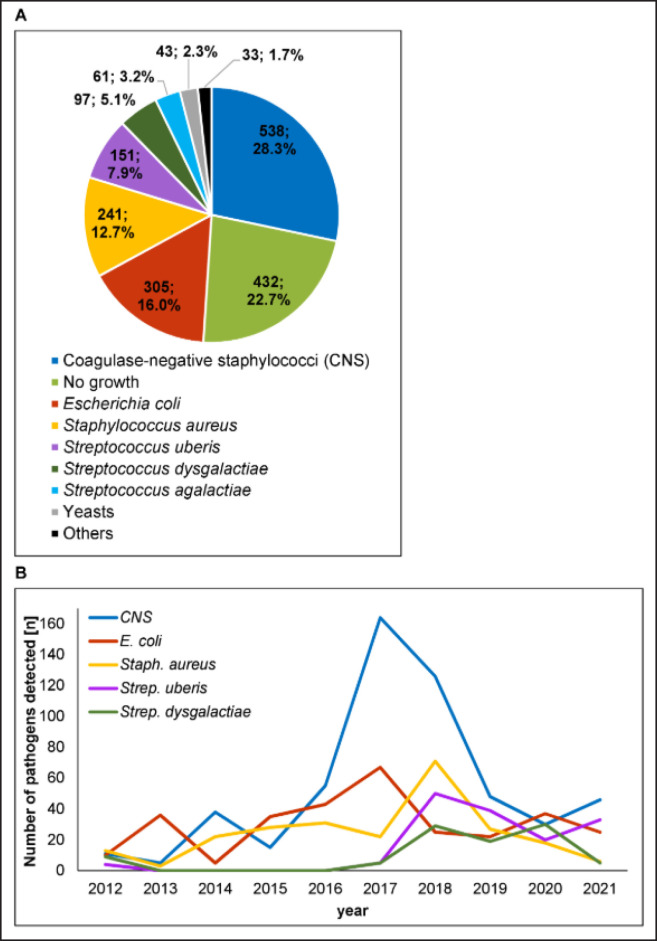
Mastitis-causing pathogens. A) distribution of 1901 bacteriological mastitis isolates (absolute number; percentage), B) detection numbers of the five most frequently determined mastitis pathogens over the monitoring period from 2012 to 2021.

In our study, we evaluated the frequency of mastitis pathogens from 1901 laboratory analyses. The highest number of mastitis cases were caused by CNS (28.3%), followed by *E. coli *(16%) and* Staph. aureus *(12.7%) (Fig. 5A). In 22.7% of milk samples from mastitis cows, no pathogen could be identified. At our analyzed farm, pathogens more rarely detected in connection with mastitis were *Strep. uberis* (7.9%), *Strep. dysgalactiae* (5.1%), *Strep. agalactiae* (3.2%), yeasts (2.3%), and others (1.7%). Over 130 pathogens are known to be associated with bovine mastitis, but >80% of all mastitis cases are induced by a limited number of predominant bacteria (*E. coli, Strep. uberis, *CNS,* Staph. aureus, Strep. dysgalactiae*, and* Strep. agalactiae) *[8,12]. Here, the most frequently identified pathogen was CNS. CNS is a group of *Staphylococcus* species, including, e.g., *Staph. chromogenes*,* Staph. simulans,* *Staph. epidermidis,* and *Staph. xylosus *[37,38]. To date, more than 20 CNS species have been identified in association with bovine mastitis. CNS are significant mastitis-causing pathogens in several countries worldwide and mostly cause subclinical infections [39]. CNS are considered environmental, rather than contagious mastitis pathogens. Therefore, our result of CNS as the leading pathogen agrees with observations that contagious pathogens were massively reduced after establishing the five-point hygienic plan in the 1960s [8,21].

Many previous studies have reported CNS as the leading cause of mastitis, especially in herds in which other major pathogens, such as *Staph. aureus* and *Strep. agalactiae*, were under control [40,41]. Infections with CNS are mostly observed immediately after calving, suggesting that the pathogen may be acquired during the dry period before calving [37]. CNS mastitis was reported to be more common in the first lactation compared to later lactations. This typical CNS characteristic of the highest mastitis numbers in the first lactation was observed in this study as well (Fig. 2A). Therefore, proper monitoring and protection of heifers is important for preventing CNS mastitis. This includes a clean and dry cow environment during the weeks before and after calving, fly control, and preventing suckling between heifers. CNS are constituents of the normal cow skin microbiota. They also colonize the teat canal and udder, with effects on udder health that may vary among CNS species [42].

A recent molecular study detected non-aureus staphylococci in 100% of cow udders and in 98% of quarter milk samples from healthy cows [43]. It is therefore possible that routine bacteriological diagnostics will occasionally blame CNS as the causative agent of mastitis, while other pathogens may be present but go undetected by the bacteriological cultivation methods applied. In agreement, for 22.7% of mastitis milk samples (432 of 1901) in our study, no causative pathogen could be identified. Reasons for that could be a high level of contamination with fecal and environmental pathogens due to an improper sampling method or mastitis causative pathogens that were not detectable with the used diagnostic approach. A high rate of no-growth and contaminated samples (27%–50%) is common for the cultivation-based diagnostic approach [44], which is applied widely due to low costs and good detection sensitivity for targeted mastitis microorganisms.

**Figure 6.**
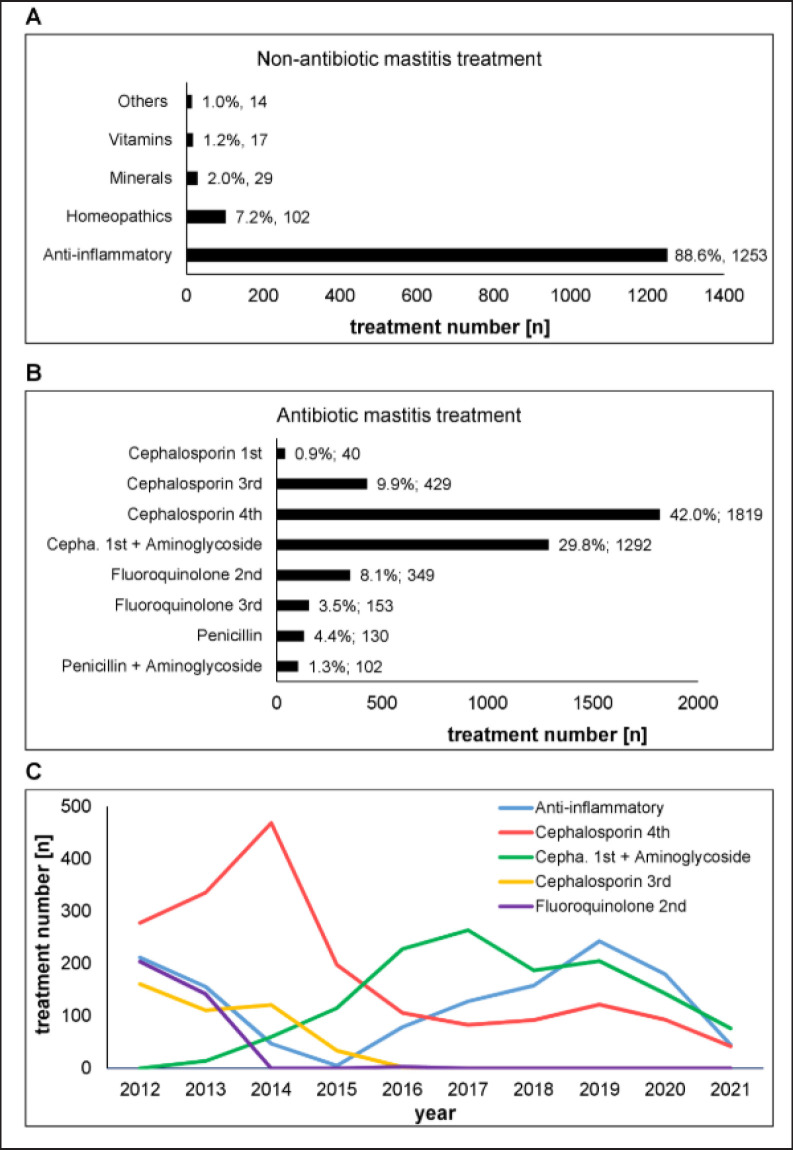
Mastitis treatment medication. Drug agents, percentage distribution, and absolute numbers of usages are shown. A) non-antibiotic mastitis treatments, B) antibiotic mastitis treatments, C) distribution of treatment numbers of the five most frequently used mastitis drugs over the monitoring period from 2012 to 2021.

When evaluating the yearly appearance of the five most frequently detected mastitis pathogens from this farm (Fig. 5B), CNS were much more often detected in 2017 and 2018, despite the number of mastitis cows not being increased within these years (36 and 33, respectively). However, the number of bacteriological analyses that failed to detect the mastitis-causing pathogen was lowest within these 2 years (data not shown), and the same cows could have several CNS infections within these 2 years. *E. coli* and *Staph. aureus* had a relatively equal distribution over the years, with minor peaks in 2017 (*E. coli*) and 2018 (*Staph. aureus*). *Step. uberis* and *Strep. dysgalactiae* were rarely detected before 2018.

**Figure 7.**
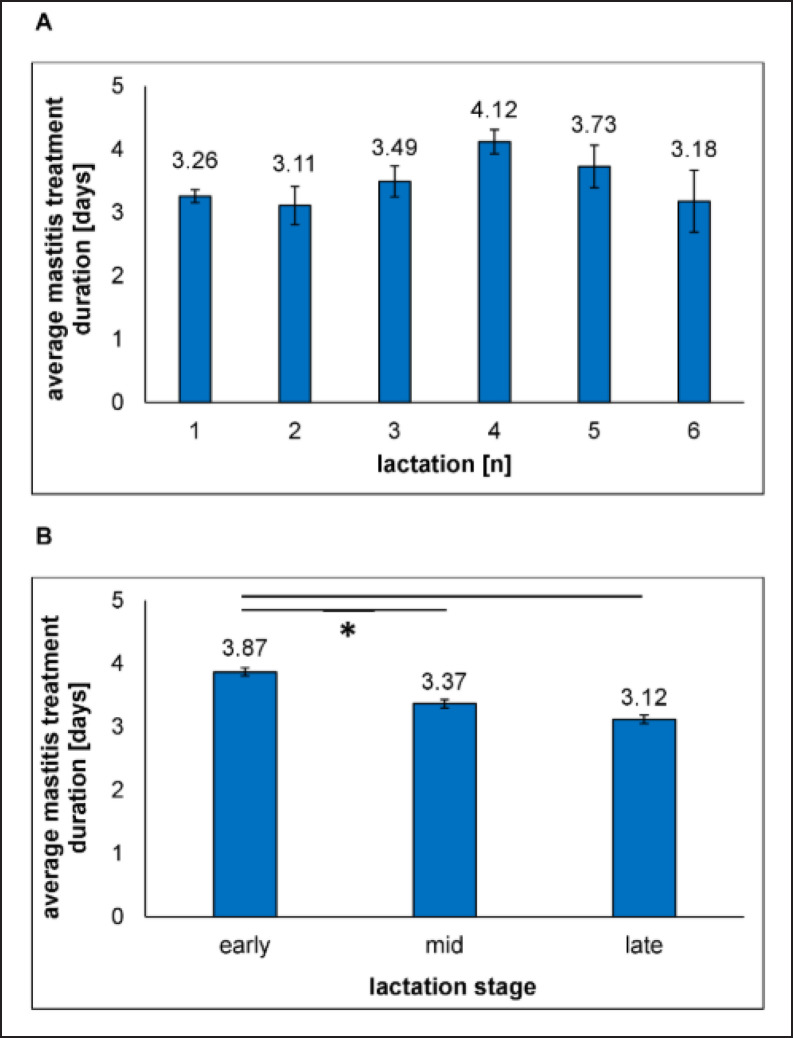
Average mastitis treatment durations according to A) lactation number and B) lactation stage in early (lactation month 1–3), mid (month 4–6) and late lactation (month ≥7). Error bars show the standard error of the mean. *indicates significant differences by Student's *t*-test (*p* < 0.05).

### Drugs and duration of mastitis treatment

Drugs used to treat mastitis infections during lactation and in the dry period (quarter selective dry cow therapy) were categorized into “non-antibiotic” and “antibiotic.” In total, 75% of all mastitis cases were treated with an antibiotic, and 25% of cases were treated with non-antibiotic drugs. In cases of a non-antibiotic treatment, anti-inflammatory medicine was the most used (88.6%, Fig. 6A). Anti-inflammatories include mainly glucocorticoids, antiphlogistics, antipyretics, and analgesics. Other alternative mastitis treatments were based on homeopathy (7.2%), minerals (2.0%), vitamins (1.2%), or others (1.0%). When an antibiotic mastitis treatment was conducted, the most used antibiotic class was fourth-generation cephalosporins (Fig. 6B, 42%). This was followed by antibiotics combining a first-generation cephalosporin with an aminoglycoside (29.8%). Fluoroquinolone and penicillin-based antibiotics were administered as well but rarely. As described, CNS mastitis was detected most frequently and can be treated with antibiotics. The overall prevalence of antibiotic resistance in CNS mastitis pathogens is reported to be low; however, for some species, such as *Staph. epidermidis*, an elevated level of β-lactamase resistance (in approx. 40% of isolates) was observed [38,39]. Resistances to more than one antibiotic have been observed for 7% and 9% of mastitis-causing CNS isolates [38].

The yearly applications of the five most frequently used mastitis drugs are shown in Fig. 6C. 2012 was the year with the highest number of mastitis cows (Fig. 1C) and the highest drug consumption over the monitoring period, and 2021 had the lowest (data not shown). The most frequently used antibiotic class, fourth-generation cephalosporins, was especially often applied in 2012-2014, with a peak in 2014. This agrees with the period with the most recorded mastitis cows (Fig. 1C). Second-generation fluoroquinolones and third-generation cephalosporins were administered only until 2013 and 2015, respectively. On 28th Feb 2018, there were legal changes in pharmaceutical drug legislation and antibiotic use by veterinarians in Germany (Verordnung über tierärztliche Hausapotheken), which does not seem to have had a main influence on the antibiotic usage of this farm.

The average length of a mastitis treatment in our study was 3.48 days (Fig. 7). This fits with the recommendations of treatment durations for most mastitis antibiotics. Analyzing treatment durations according to the number of lactations did not deliver major variations. Dairy cows in the fourth lactation got the longest treatments of approx. 4.12 +/− 0.19 days, and the shortest treatment durations were observed for cows in the second lactation (3.11 +/− 0.23 days). This results in a treatment length variation of a maximum of 24.2 h or 24.5%, which probably represents the application of one drug dosage more or less. When separating mastitis treatment durations by lactation stage, cows in the early lactation needed the longest treatments of 3.87 +/− 0.06 days, and animals within the late lactation got the shortest treatments (3.12 +/− 0.05 days). A significant difference (*p* < 0.05) in treatment duration was detected for early lactating cows, compared to middle and late lactating cows. Here, a variation in the average treatment length of a maximum of 20.2 h or 21.7% was observed.

## Conclusion

This study analyzed the mastitis situation, including frequencies, pathogens, and treatments, of a commercial dairy AMS farm in Germany over a remarkably long period of 9 years. This case report gives detailed insights into the mastitis situation under practical production conditions and includes a resilient amount of data. Especially the information about mastitis recurrence, drug usage, and duration, as well as the change after AMS installation, are rarely described previously and are relevant. We identified factors with elevated risks for mastitis (summer season, first and >7 lactations, and late lactation stage) and delivered information to deduce better mastitis prevention measures. This will be a valuable information source and improve our knowledge about the severity of mastitis in the commercial production system.

## List of abbreviations

AMS, Automatic milking system; CNS, Coagulase-negative staphylococci; SCC, somatic cell count.
